# A diagnostic approach for differentiating abdominal tuberculosis from ovarian malignancy: a case series and literature review

**DOI:** 10.1186/s12919-019-0180-y

**Published:** 2019-12-16

**Authors:** Moh Nailul Fahmi, Annisaa Pelita Harti

**Affiliations:** Department of Obstetrics and Gynecology, Faculty of Medicine, Public Health, and Nursing, Universitas Gadjah Mada/Dr. Sardjito Hospital, Jl. Kesehatan No. 1, Yogyakarta, 55281 Indonesia

**Keywords:** Abdominal tuberculosis, Ovarian mass, Diagnosis, Laparotomy

## Abstract

**Background:**

Abdominal tuberculosis is an uncommon variant of extrapulmonary tuberculosis. It accounts for 3.5% of extrapulmonary tuberculosis. Diagnosis of abdominal tuberculosis is still a challenge due to its non-specific symptoms. Abdominal tuberculosis and ovarian cancer may show similar symptoms, laboratory and imaging features. The goal of our report is to emphasize for the need of a diagnostic approach based on clinical manifestations, laboratory, imaging findings, and additional tests for considering a diagnosis of abdominal tuberculosis rather than ovarian cancer.

**Case presentation:**

We report 3 cases of abdominal tuberculosis in our Onco-gynaecology Division, Department of Obstetrics and Gynaecology, Sardjito Hospital, Yogyakarta, Indonesia in 2018 which were previously diagnosed as ovarian malignancy and managed surgically. All of our patients experienced abdominal pain and enlargement but only two of them had significant weight loss. The general symptoms were typically found in onco-gynaecology patients, especially in those with ovarian malignancy. Ultrasound examination showed multilocular masses, 2 of them with solid parts and ascites. Cancer antigen 125 (CA-125) levels were found increasing in those three patients. All of them were treated surgically and diagnosis of abdominal tuberculosis was established through the histopathological result of tissue biopsy. Based on our cases and literature, we consider the need of a diagnostic approach to differentiate abdominal tuberculosis from ovarian malignancy, an attempt to avoid unnecessary invasive procedures that put burden risk for the patients.

**Conclusion:**

Minimally invasive tests to establish the diagnosis of abdominal tuberculosis should be optimized to reduce the burden risk of laparotomy. Careful diagnostic steps should be followed to avoid wrong diagnosis.

## Background

Tuberculosis is an infectious disease caused by *Mycobacterium tuberculosis*, which mostly affects the lung, but can also affect other organs, referred as extra-pulmonary tuberculosis [1]. Abdominal tuberculosis contributes about 3.5% of extra-pulmonary cases. Abdominal focuses of mycobacterium were the result of hematogenous spread from primary pulmonary focuses, or may also be caused by swallowed bacilli which transported through lymphatic by macrophage to the mesenteric lymph nodes [2].

The most common presenting symptoms were abdominal pain, weight loss, fever, abdominal mass, and ranges of another symptoms including vomiting, constipation, abdominal tenderness, and signs of ascites and peritonitis [3]. Abdominal tuberculosis frequently shares common symptoms with ovarian malignancy. Several laboratories and imaging modality are often utilized in attempt to distinguish between those two. In some patient surgery was performed on indication of ovarian tumor due to similarity of physical examination and imaging result. Diagnostic approach is needed to eliminate unnecessary laparotomy due to wrong diagnosis.

## Case presentation

Three cases of abdominal tuberculosis previously diagnosed as ovarian malignancy were identified in our Onco-gynecology Division, Department of Obstetrics and Gynecology, Dr. Sardjito Hospital during year 2018. All of them were treated surgically and diagnosis of abdominal tuberculosis was established through histopathological result of tissue biopsy. The summary of each case is presented below (Table 1).

**Table 1:** Characteristic of each patient

	Patient 1	Patient 2	Patient 3
Age	16 years old	16 years old	32 years old
Parity	P0A0	P0A0	P1A0
Body mass index	24.06 kg/m2	15.80 kg/m2	14.70 kg/m2
Main complains	Abdominal pain and enlargement	• Abdominal pain and enlargement • Nausea and vomiting • Weight loss	• Abdominal pain and enlargement • Weight loss
Ultrasound	A cystic mass in left adnexa, measured 43 × 37 mm, with solid parts and irregular border with ascites.	A large multilocular abdominal mass filled the pelvic cavity, without ascites.	A large multilocular abdominal mass, with solid parts, highly vascularized, with large amount of ascites fluid.
CT scan	• A complex left ovarian cyst with loculated ascites, suggesting malignant appearance. • Bilateral inguinal lymphadenopathy. • Marked thickening of peritoneum.	Not performed	• A multilocular cyst from left and right adnexa along with marked ascites. • There is no paraaortic, mesenteric, and iliac lymph nodes enlargement. • Smooth thickening and enhancement of peritoneum
CA-125	886 U/mL	481 U/mL	203 U/mL

### Case 1

A 16 years old female was referred from district hospital. Her main complaints were abdominal pain and enlargement for the last 2 months. The suspicion of malignant ovarian cyst was established from referring obstetrician based on abdominal ultrasound. Defecation and micturition pattern were normal.

Her menstrual cycle was normal, with 28–30 days’ cycle and 4–5 days of menstrual period in each cycle. There was no history of fever, vaginal discharge, chronic illness, chronic cough, and significant weight loss. There was no obvious contact with person with tuberculosis or those in tuberculosis therapy.

A thorough physical examination revealed slightly distended abdomen, with palpable cystic mass up to 2 cm above pubic symphysis. From bimanual palpation, uterus was palpable within normal size, with palpable cystic mass in left adnexa.

Abdominal ultrasound showed a cystic mass in left adnexa, measured 43 × 37 mm, with solid parts and irregular border, along with peritoneal free fluid. Abdominal computed tomography (CT) scan further showed a complex left ovarian cyst with ascites, suggesting malignant appearance. CT also founded right renal pelviectasis, hepatosplenomegaly, and bilateral inguinal lymphadenopathy.

Laboratory workup for tumor biomarker was performed, with result supporting the suspicion of malignancy process (CA-125: 886 U/mL).

Exploratory laparotomy was performed to found the fragile, solid mass which filled most of abdominal cavity and adhered to the pelvic wall, a condition commonly known as ‘frozen pelvis’, causing further exploration without making massive tissue destruction was impossible. Operator decision was to close the abdomen after collecting some tissue for histopathology workup.

**Fig. 1:**
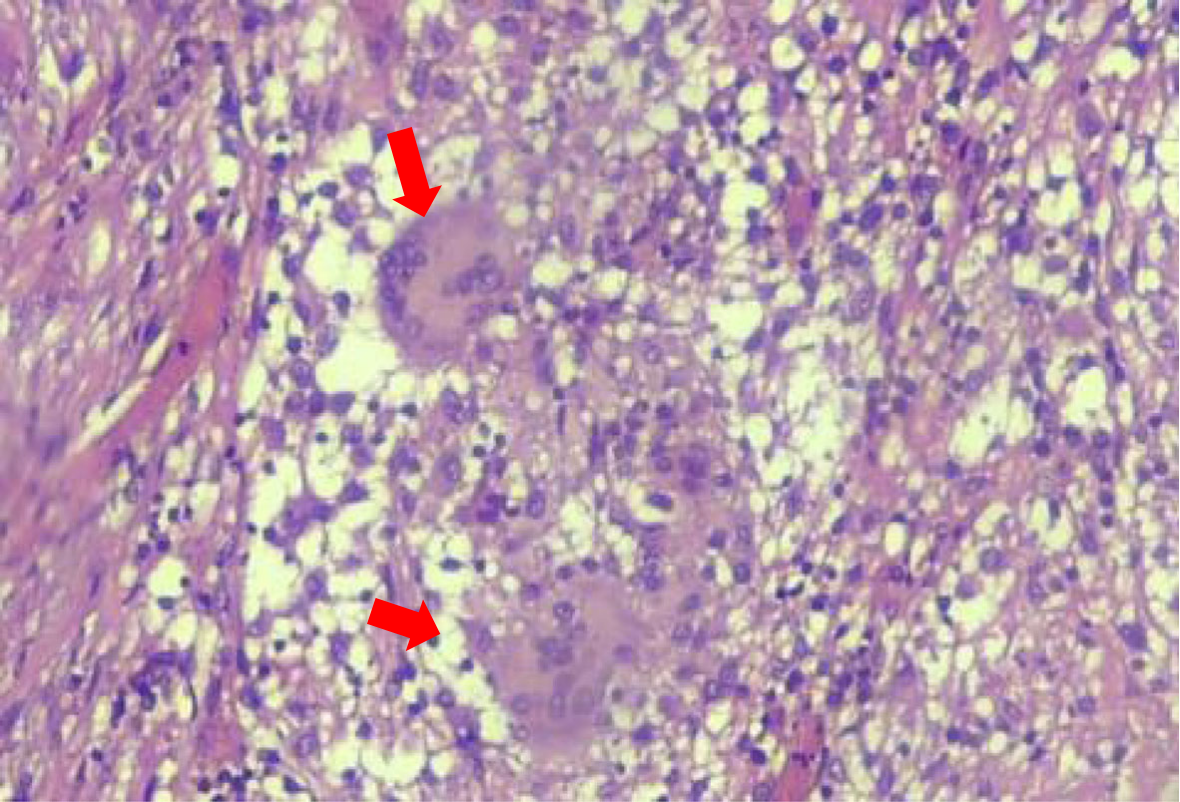
Specific granulomatous process defined by pathognomonic multinucleated giant cells (red arrows) surrounded by abundant lymphocytes. This histopathological slide is taken from laparotomy and peritoneal biopsy of patient 1

Histopathology report came out a week later, revealing a granulomatous inflammation related to tuberculosis process (Fig. 1). The diagnosis of abdominal tuberculosis was subsequently established.

### Case 2

A 16 years old female was referred from a private local hospital with suspected ovarian malignancy. She reported painful abdominal enlargement since the last year along with nausea and vomiting and marked weight loss. No history of fever, chronic cough, nor contact with tuberculosis-positive persons. Previous ultrasound examination showed ovarian mass with malignant appearance.

She appeared cachexic, with body mass index only 15.8 kg/m2. Abdominal palpation revealed a lower abdominal mass originating from pelvic cavity up to umbilical level. Abdominal ultrasound showed large multilocular abdominal mass filling the pelvic cavity. No ascites fluid was found. Unfortunately, abdominal CT scan was not performed for this patient. Tumor marker was checked and CA-125 was found high (481 U/ml).

She was diagnosed with suspected ovarian malignancy and laparotomy was planned. During surgery, parietal peritoneum was found thick and easily bleed. After it was opened, massive adhesion of abdominal organ was found and further exploration was considered impossible without damaging surrounding organs. Surgery was completed after collecting peritoneal tissue to be sent to pathology laboratory.

Histopathological result showed granulomatous inflammation specific for tuberculous infection. The patient then was sent to internal department to receive extrapulmonary tuberculosis drug regimen.

### Case 3

A 32 years old female was referred from internal department with suspected ovarian malignancy. She felt painful abdominal enlargement since the last 4 months due to massive ascites. Abdominal paracentesis had been done twice to reduce the ascitic fluid and temporarily eliminated the symptoms. Ascites fluid culture showed numerous Gram-positive coccus and Gram-negative bacillus, but acid-fast staining was not performed. Lower abdominal ultrasound ordered by internal department revealed abdominal cystic mass from ovarian origin.

The patient experienced significant weight loss for the last 4 months with body mass index 14.7 kg/m2. A large abdominal cystic mass was palpable through physical examination and confirmed by abdominal ultrasound examination. The mass was multiloculated, with solid parts and highly vascularized. Significant amount of ascites was seen.

Abdominal CT scan showed small multilocular cyst from left and right adnexa along with marked ascites. There is no paraaortic, mesenteric, and iliac lymph nodes enlargement. Abdominal paracentesis was done, and culture workup showed marked negative Gram-staining bacilli. Acid-fast stain was not performed. Tumor marker for epithelial ovarian malignancy was rising (CA-125: 203).

The patient was suspected to have ovarian malignancy and planned to have laparotomy procedure. During procedure, peritoneal cavity was filled with yellowish caseous necrotic tissue pathognomonic for tuberculous process, forming a cystic-like mass. Four liters of the tissue was evacuated and was sent for culture and cytology workup. Peritoneal biopsy was done, and no further exploration was performed because of massive adhesion. The result came out a week later, all confirmed tuberculous infection.

## Discussion

The most common presenting symptoms of abdominal tuberculosis are abdominal pain (95%), followed by weight loss (88%), fever (84.6%), abdominal mass (46.1%) and ranges of another symptoms including vomiting, constipation, abdominal tenderness, and signs of ascites and peritonitis [3]. Meanwhile, increased abdominal size or bloating, urinary urgency, difficulty eating and abdominal/pelvic pain are often reported by patients with ovarian malignancy [4].

All our patients experienced abdominal pain and enlargement but only two of them had significant weight loss. The general symptoms were typically found in onco-gynecologic patients, especially in those with ovarian malignancy. All of them were referred to our department because of suspected ovarian-origin mass showing malignancy signs. Theoretically, abdominal tuberculosis frequently shares common symptoms with ovarian malignancy. Several laboratories and imaging modality are often utilized in attempt to distinguish between those two.

Tumor marker CA-125 was not useful to distinguish abdominal tuberculosis from ovarian malignancy. Numerous case reports and case series showed significantly elevated CA-125 in patient diagnosed with abdominal tuberculosis [5–8]. Similarly, increased level of CA-125 was found in all our patient. Meanwhile, decrease of serum CA-125 in patient with abdominal tuberculosis receiving course of anti-tuberculosis drug indicating some value of this biomarker in evaluation of tuberculosis treatment [9].

Human epididymis protein 4 (HE4) belong to four-disulfide family of protein and normally act as proteinase inhibitor. Highest expression on HE4 was observed in ovarian malignancy, especially serous and endometrioid adenocarcinoma [10, 11]. The risk of ovarian malignancy algorithm (ROMA) utilizing both CA-125 and HE4 level is comparable to risk of malignancy index (RMI) as diagnostic tool to differentiate ovarian malignancy [12, 13]. HE4 was also found to rise in pulmonal tuberculosis, but its role in detecting abdominal tuberculosis is less understood. A retrospective study found that serum HE4 in peritoneal tuberculosis was significantly lower than that in ovarian malignancy. An optimal cut-off value, 151.4 pmol/l, was established to differentiate between those two [14].

**Fig. 2:**
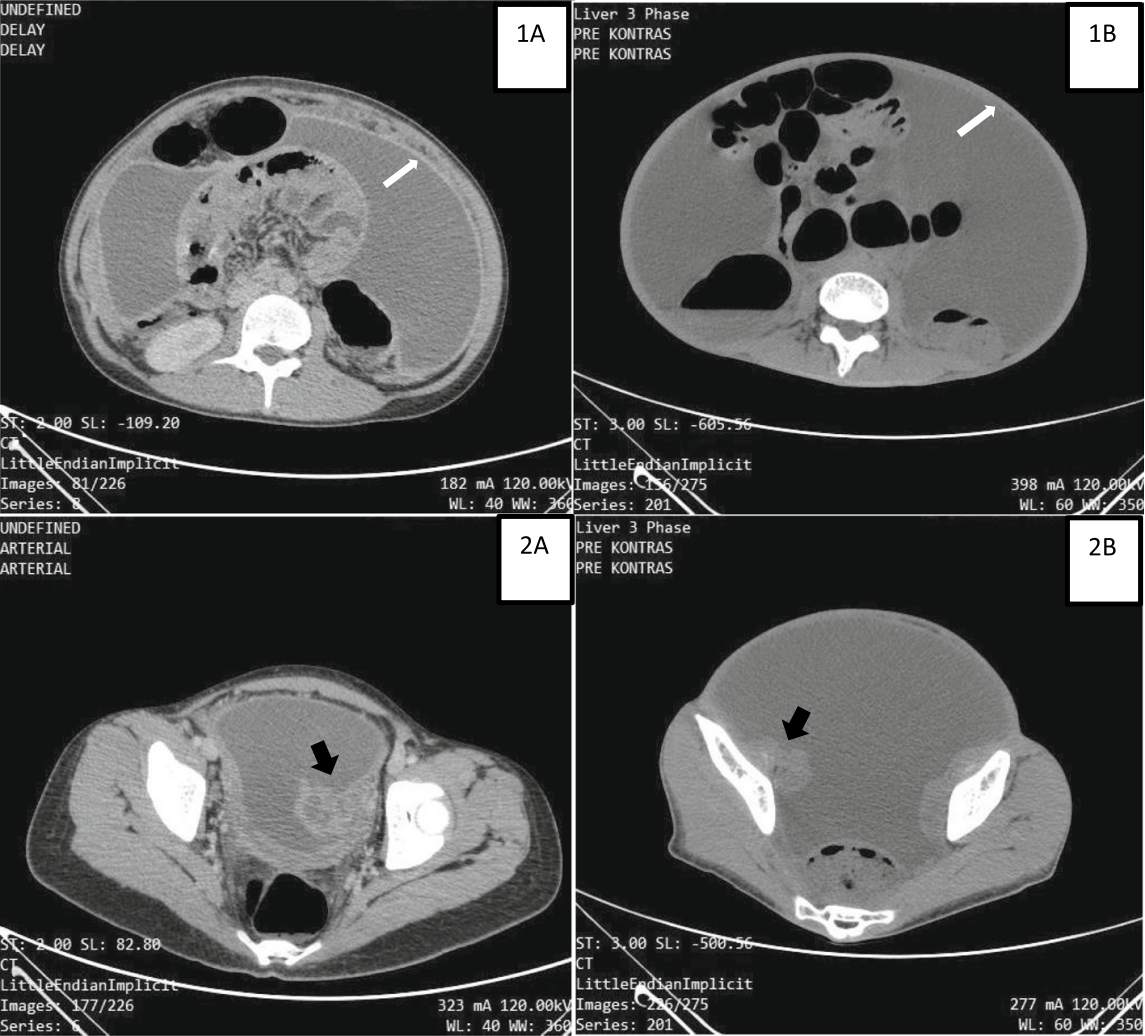
Abdominal CT scan in Patient 1 and Patient 2. Both showed significant amount of ascites (white stars) with smooth non-nodular parietal peritoneum thickening (white arrows). Hypo-isodense multiloculated cystic mass of adnexa previously interpreted as ovarian tumor (black arrows) in Patient 1 and 2

In our cases, 3 patients came with ultrasound examination showing multilocular mass, 2 of them with solid parts and ascites. These 2 patients were considered to require further imaging workup, so abdominal multiple CT scan with contrast was ordered (Fig. 2).

The first patient had complex left ovarian cyst with loculated ascites, thus suspected as ovarian malignancy. In this patient, bilateral inguinal lymph nodes enlargement and marked peritoneal thickening was found.

The third patient was suspected with ovarian malignancy due to appearance of multiloculated cystic mass originated from ovary that infiltrate uterine tissue along with ascites and smooth peritoneal thickening. There was no lymph nodes enlargement.

Abdominal scan was commonly used imaging in patient with suspected ovarian mass. In a study describing CT scan result of 10 patients with confirmed abdominal tuberculosis, omental and mesenteric thickening along with ascites were found in all patients, while cystic ovarian mass or enlargement and peritoneal implants were not consistently seen [15]. The parietal peritoneum involvement in CT seemed to have diagnostic value, in which most of patients with abdominal tuberculosis will have smooth thickening of parietal peritoneum while irregular and nodular involvement was commonly found in peritoneal carcinomatosis [16]. Although ultrasound and CT scan have been considered to be reliable in diagnosis of abdominal tuberculosis [17], in many cases it failed to distinguish abdominal tuberculosis from ovarian malignancy [5, 18].

Several tests were available to detect mycobacterium from ascites. Unfortunately, none of them was performed to our patient because abdominal tuberculosis was not suspected at the first place.

Polymerase chain reaction (PCR) of ascetic fluid for mycobacterium can be considered for diagnosis and should at least be attempted before surgical intervention, but this technique is not widely available [5]. Furthermore, reports suggest that the although performance of the various PCR tests is reasonably good with sensitivity reaching up to 95% in smear-positive patients, the same success has not been duplicated in smear-negative patients and the sensitivity attained has been disappointingly low (48%) [19].

The X-pert MTB/RIF assay is a nucleic acid amplification test that is reliable to diagnose tuberculosis diseases and drug resistance rapidly. Its use now is recommended as the preferred initial test to establish the diagnosis of tuberculous meningitis [20]. However, utilization of this method for ascites fluid analysis to diagnose abdominal tuberculosis is still questioned due its poor sensitivity [21, 22].

Adenosine deaminase (ADA), purine degrading enzyme, is widely distributed in tissue and body fluid. ADA is necessary for T lymphocytes proliferation and differentiation, a prominent process in immune cellular response against M. tuberculosis. A meta-analysis found that ADA level of ascites fluid above 39 IU/L was reliable to diagnose peritoneal tuberculosis with 100% sensitivity and 97.2% specificity [23]. This finding was supported by numerous study [16, 18, 24].

T-cell-based interferon gamma release assay (IGRA) was considered as a substitute for tuberculin skin test with higher sensitivity and specificity to detect mycobacterium infection [25]. Nevertheless, suboptimal result is possible due to its inability to distinguish latent infection from active diseases. A meta-analysis involving 1711 patients with blood samples to determine the accuracy of IGRA in diagnosis of extrapulmonary tuberculosis showed sensitivity between 72 and 90%, while the specificity ranged between 68 and 82% (depending on various IGRA test commercially available) [26]. While the sensitivity and specificity of IGRA is lower than ADA test, it gives diagnostic advantages because invasive procedure to obtain the ascites fluid is not necessary.

Laparoscopy is an important tool in the management of such cases to avoid extended surgery. While visual diagnosis using this minimally invasive technique was highly accurate, mycobacteria was only scarcely found on histological sections [27]. This is due to paucibacillary nature of tuberculous peritonitis, making the classical method of Ziehl-Neelsen stain and mycobacterium culture from ascetic fluid or peritoneal biopsy such a poor diagnostic tools [28].

Utilizing Xpert MTB/RIF from tissue samples could be another alternative. The pooled estimate of sensitivity was calculated as 81.2% (95% CI, 67.7–89.9%) while the pooled specificity was 98.1% (95% CI, 87.0–99.8%) compared to tissue culture [29].

Based on our cases and above-mentioned studies and literatures, we consider the need of a diagnostic approach to differentiate abdominal tuberculosis from ovarian malignancy as we propose below (Table 2). Patient with history, physical examination, ultrasound, and abdominal CT scan suggestive of abdominal tuberculosis deserve further evaluation to differentiate between those two conditions in order to avoid unnecessary invasive procedure.

**Table 2:** A diagnostic approach to differentiated abdominal tuberculosis from ovarian malignancy

	Abdominal Tuberculosis	Ovarian Malignancy
Chief complains	Symptoms may present in both diseases	
	abdominal pain, weight loss, abdominal mass, bloating, constipation, difficulty eating, signs of ascites [2, 3].	
	Specific symptoms	
	fever (84.6%)	–
Physical examination	Common physical examination results of both diseases	
	• abdominal mass • ascites • abdominal tenderness • weight loss (underweight) [30]	
	No single specific physical examination to differentiate abdominal TB and Ovarian Malignancy), following signs tend to be presented in one disease, but can be found in the other under specific condition	
	• Solid organ enlargement (hepatomegaly, splenomegaly, or hepatosplenomegaly) • Inguinal lymphadenopathy	• Localised adnexal mass (in early stage) • Pleural effusion (advanced stage) • Liver metastasis (advanced stage) [31]
Abdominal Ultrasound	Common	
	Cystic mass	
	Specific	
	• Ascites (free or loculated, clear or complex with membranes, septum, or debris) • Peritoneal or omental thickening • Lymph node involvement (periportal, peripancreatic, mesenteric, or retroperitoneal • Bowel wall thickening or distended fluid-filled bowel loops. • Abdominal abscesses • Visceral involvement: homogeneous organomegaly, focal lesion, or calcified foci [32]	• Presence of ascites • Peritoneal masses (nodular), enlarged nodes, or matted bowel [33] • Solid part that is often nodular or papillary • Irregular, thick septations • Color or power Doppler demonstration of flow in the solid component [33].
Abdominal CT scan	Common	
	Cystic mass	
	Specific	
	• Free or loculated ascites • Smooth thickening of the peritoneum • Lymph nodes enlargement with central necrosis and calcification • Thickening of the mesentery and omentum • Homogenous organomegaly [34]	• Primary ovarian mass • Multinodular and irregular peritoneal thickening • Homogeneous retroperitoneal lymph nodes enlargement • Omental cake • Hepatic and splenic focal metastatic lesion [34, 35]
Common additional tests		
CA-125	Increased [7–9, 15]	Increased [12, 13]
HE4	Increased (≤151.4 pmol/l) [14]	Markedly increased (>151.4 pmol/l) [10, 12–14]
Specific Additional tests		
Specific Additional tests	• Polymerase chain reaction for mycobacterium of ascites fluid [18, 36] • Xpert MTB/RIF assay of sputum or tissue biopsy [20, 29] • Amino deaminase test of ascites fluid [23, 24] • T-cell-based interferon gamma release assay (IGRA) of ascites fluid or blood [25] • Visual diagnostic using laparoscopy approach. (thickened peritoneum with yellowish-white lesions, with or without adhesions, fibroadhesive pattern) [23] • Culture or histopathology examination of peritoneal biopsy (as gold standard either by laparoscopy or laparotomy) [23, 24, 27]	• Imaging for metastatic diseases (Magnetic resonance imaging, thorax X-ray, positron emission tomography) [37] • Paracentesis, thoracentesis, image-guided biopsy [38, 39] • Surgical evaluation

From the recent study, adenosine deaminase assay of ascites fluid gives the best accuracy in diagnosing abdominal tuberculosis due its high sensitivity and specificity. IGRA test using blood sample can be the alternatives in such case where invasive procedure cannot be performed. We propose to do ADA test and IGRA test in case ultrasound or CT scan findings were suggestive for abdominal TB. If ADA test with or without IGRA test is positive then we may consider managing the patients as abdominal tuberculosis.

Laparoscopy is preferred procedure over exploratory laparotomy, not only does it allow the inspection of the peritoneum but also offers the option of obtaining specimens for histology, while giving lower risk of surgical morbidity. Despite of attempts to make it minimally invasive, more than half of patients need laparotomy to established diagnosis of abdominal tuberculosis [40].

## Conclusions

In addition to ovarian cancer, the diagnosis of abdominal tuberculosis should always be considered in patients with abdominal distension, pain, weight loss, and signs and symptoms of ascites, especially in an endemic area of tuberculosis. Careful diagnostic steps should be followed to avoid the wrong diagnosis. Minimally invasive procedures should be optimized to reduce the burden risk of laparotomy. Exploratory laparotomy could be performed to establish a diagnosis of abdominal tuberculosis to rule out ovarian malignancy when the standard tests were negative.

## Data Availability

All data generated or analysed during this study are included in this published article.

## Abbreviations

ADA: Amine deaminase
CA 125: Cancer antigen 125
CT: Computed tomography
HE4: Human epididymis protein 4
IGRA: Interferon gamma release assay
PCR: Polymerase chain reaction
